# Effects of the histone deacetylase inhibitor valproic acid on Notch signalling in human neuroblastoma cells

**DOI:** 10.1038/sj.bjc.6602309

**Published:** 2005-02-01

**Authors:** M-T Stockhausen, J Sjölund, C Manetopoulos, H Axelson

**Affiliations:** 1 Department of Laboratory Medicine, Division of Molecular Medicine, Lund University, University Hospital MAS, S-205 02 Malmö, Sweden

**Keywords:** VPA, TSA, Notch, Hes-1, Hash-1, proliferation, differentiation, neuroblastoma

## Abstract

Neuroblastoma (NB), a sympathetically derived childhood tumour, shows characteristics of neuronal precursor cells, suggesting a halted differentiation process. We have previously shown that the Notch signalling cascade, a key player during normal neurogenesis, also might be involved in NB differentiation. Valproic acid (VPA), a well-tolerated antiepileptic drug, has been shown to induce differentiation and cell death of NB cells, possibly associated with its recently described HDAC inhibiting activity. Stimulation of NB cells with VPA led to increased cell death and phenotypic changes associated with differentiation, that is, neurite extension and upregulation of neuronal markers. VPA treatment also led to an activated Notch signalling cascade as shown by increased levels of intracellular Notch-1 and Hes-1, mimicking the initial phase of induced differentiation. These results reinforce that VPA potentially could be used in differentiation therapy of NB and that the effects in part could be a consequence of interference with the Notch signalling cascade.

The paediatric tumour neuroblastoma (NB) is thought to arise due to perturbed differentiation during development of the sympathetic nervous system (SNS). The cells express molecular markers transiently expressed during sympathetic development, for example, the basic helix–loop–helix (bHLH) proteins Hash-1 and dHAND (Gestblom *et al*, 1999; Grynfeld *et al*, 2000). Spontaneous regression of a specific subclass of NB, that is, stage 4S, is known to occur and is a result of differentiation of the tumour cells into benign ganglioneuroma (Brodeur *et al*, 1997). The overall survival rate in NB is low, around 40–50%, and most often the children are treated with a combination of cytostatics, with frequent relapses of tumour growth.

The Notch signalling cascade is important in the development of several tissues, including the peripheral and central nervous systems (Artavanis-Tsakonas *et al*, 1999). Notch signalling is controlled by local cell–cell interactions based on the expression of Notch receptors and ligands on neighbouring cells that activate the cascade. This activation most often results in downregulation of tissue-specific transcriptional activators and the cells are retained in an undifferentiated state. However, the cellular response to Notch signalling is highly cell type specific and in some cases it also triggers a differentiation process (Rangarajan *et al*, 2001; Sriuranpong *et al*, 2001; Schroeder *et al*, 2003). When Notch binds to its ligand, Delta-like and Jagged in humans, it goes through two consecutive cleavages at specific sites: S2 and S3. The S2 cleavage that occurs just outside the cell membrane is performed by ADAM-like proteases and exposes the S3 cleavage site (Val 1744) situated in the transmembrane region. S3 cleavage is conducted by a *γ*-secretase complex containing presenilin and results in an activation of the Notch protein by releasing the intracellular part of the molecule (i.c. Notch), which is then transported to the nucleus (Mumm and Kopan, 2000). In the nucleus i.c. Notch binds to RBP-J*κ* (also referred to as CSL and CBF-1), which in its quiescent state, binds to co-repressors mediating a histone deacetylase (HDAC) activity on Notch target genes (Kao *et al*, 1998). When i.c. Notch binds to RBP-J*κ* it displaces HDAC and the co-repressors and instead transcriptional co-activators are recruited. One such co-activator is the mastermind like proteins (MAML), which mediate the interaction with basal transcriptional activators, such as CBP/p300 (Petcherski and Kimble, 2000). Thus, the transcriptional activity of RBP-J*κ* is not only regulated by complex formation with Notch but also requires expression of several co-factors.

One direct and important transcriptional target of Notch signalling is the bHLH protein Hairy and Enhancer of Split-1 (Hes-1) that functions as a negative regulator of transcription (Iso *et al*, 2003). Hes-1 binds the promoter region of target genes either as a homodimer or as a heterodimer together with E proteins and represses transcription by recruiting Groucho and HDAC through its N terminal WRPW region (Grbavec and Stifani, 1996; Jimenez *et al*, 1997). Hes-1 can also bind to its own promoter, and repress transcription. One important Hes-1 target gene in neuronal cells is Mammalian *achaete-scute* homolog-1 (Mash-1, Hash-1 in humans) (Chen *et al*, 1997; Grynfeld *et al*, 2000). Consequently, Mash-1 is upregulated in Hes-1 knockout mice and the animals exhibit premature neuronal differentiation (Sasai *et al*, 1992). Thus, Notch signalling is instrumental for proper temporal and spatial neuronal differentiation. During induced differentiation of NB cells we have observed a transient upregulation of Hes-1 accompanied by a downregulation of Hash-1 (Grynfeld *et al*, 2000). Furthermore, enforced expression of i.c. Notch-1 inhibits the differentiation process (Franklin *et al*, 1999; Grynfeld *et al*, 2000). Based on these results we postulate that precise temporal regulation of the Notch signalling cascade is an integral part of NB cell differentiation from highly proliferative neuroblasts into terminally differentiated neuronal cells.

Several lines of evidence indicate that aberrant acetylation of chromatin can contribute to tumorigenesis (Johnstone, 2002). This is thought to lead to transcriptional dysregulation of genes that are involved in important aspects of tumorigenic transformation, such as differentiation, proliferation and apoptosis. In some tumour forms, tumour suppressor genes are silenced partly through deacetylation of promoter regions and treatment with HDAC inhibitors therefore contribute to re-expression of these genes. A detailed understanding of the molecular mechanisms behind the often dramatic effects of HDAC inhibitors on tumour cells remains unclear.

Valproic acid (VPA) is currently used for long-term treatment of epilepsy in both adults and children. During recent years it has become evident that the drug also possesses antitumoural activity, which has led to several preclinical studies showing that VPA induces loss of proliferative capacity and promotes differentiation of several tumour cell types (Blaheta and Cinatl, 2002). The mechanisms behind these effects are still not clear, but might involve the ERK-signalling cascade, cell-cycle regulatory proteins, inhibition of protein kinase C or activation of PPAR*γ* (Yuan *et al*, 2001; Blaheta and Cinatl, 2002; Gurvich *et al*, 2004). One important finding is however that VPA functions as an HDAC inhibitor, possibly by binding to the catalytic centre of HDACs (Gottlicher *et al*, 2001). Furthermore, it was shown that VPA induced differentiation of F9 teratocarcinoma and AML cells. In addition, VPA delayed tumour formation in mouse xenograft experiments (Gottlicher *et al*, 2001). VPA has also been shown to suppress the malignant phenotype of NB cells, indicated by upregulation of some neuronal markers and decreased cell growth (Cinatl *et al*, 1997, 2002; Yuan *et al*, 2001).

In light of the finding that VPA inhibits HDAC activity, we have studied the effects of the drug on the Notch signalling cascade in NB cells, since this cascade is dependent of HDAC activity and seem to play a role in NB cell differentiation. We here report that VPA induces a more mature phenotype of NB cells along with an increased activity of the Notch signalling cascade.

## MATERIALS AND METHODS

### Cell culture

The human NB cell lines SH-SY5Y, SK-N-BE(2) and KCN-69n were grown in Eagle's minimum essential medium (Invitrogen Inc., Carlsbad, CA, USA) supplemented with 10% foetal calf serum (FCS), 100 IU/ml penicillin and 100 *μ*g ml^−1^ streptomycin in an atmosphere of 5% CO_2_. The NB cell line SH-EP derived from SK-N-SH was grown in the same media and antibiotics as above but with 15% FCS. IMR-32, LA-N-1, LA-N-2 and LA-N-5 NB cell lines were cultured in RPMI 1640 with 10% FCS and antibiotics as above. The rat pheochromocytoma cell line PC12 was grown in RPMI 1640 supplemented with 10% horse serum, 5% FCS and the antibiotics as above. SH-SY5Y and SK-N-BE(2) cells were treated with indicated concentrations of 2-propylpentanoic acid (VPA) (Sigma, St. Louis, MO, USA) or Trichostatin A (TSA) (Sigma) for indicated times before harvest. In control experiments equal volume of solvent was added, that is, methanol or DMSO, respectively. For inhibition of *γ*-secretase activity, L-685,458 (Bachem) was used at a concentration of 2.5–5 *μ*M.

### Western blot analysis

Total cell homogenates were prepared by lysing cell pellets in NP40 lysis buffer (1% NP40, 10% glycerol, 20 mM Tris-HCl pH 8.0, 137 mM NaCl and 4% complete protease inhibitor coctail mix (Roche, Mannheim, Germany)). In total, 25–50*μ*g protein per lane was subjected to SDS–PAGE, followed by blotting to PVDF (Immobilon, Millipore Corp., Billerica, MA, USA) or Hybond C (Amersham, Life Science, Solna, Sweden) filters. The filters were probed with polyclonal antiserum directed against the C-terminal part of Notch-1 diluted 1 : 50 to 1 : 100 (#sc-6014, Santa Cruz Biotechnology, Santa Cruz, CA, USA), polyclonal antiserum directed against the cleaved form of Notch-1 (Val 1744, Cell Signaling) diluted 1 : 500, polyclonal anti-Hes-1 antiserum diluted 1 : 8000 (kindly provided by Dr Tetsuo Sudo, Japan), monoclonal anti-Mash-1 antibody diluted 1 : 125 (Pharmingen, San Diego, CA, USA) or monoclonal anti-GAPDH antibody diluted 1 : 500 (Labora Chemicon International, Temecula, CA, USA). The blots were then incubated with secondary antibodies: rabbit anti-goat 1 : 2000 (Dako A/S, Glostrup, Denmark), donkey anti-rabbit 1 : 1000 (Amersham), goat anti-mouse 1 : 7000 (Jackson, West Grove, PA, USA) or sheep anti-mouse 1 : 5000 (Amersham), all coupled to horseradish peroxidase. Immunoreactivity was detected using the enhanced chemiluminescence method (Pierce). For detection of acetylated histones, cells were treated with 1 mM VPA or the corresponding volume of methanol for 12 h. Acidic cell lysates were separated by SDS–PAGE and transferred to a Hybond C nitrocellulose membrane (Amersham). Acetylated Histone H3 was then detected using a polyclonal antiacetyl Histone H3 (Lys9) antisera diluted 1 : 500 (Upstate) and a goat anti-rabbit secondary antibody 1 : 2000 coupled to horseradish peroxidase (Dako). Immunoreactivity was detected using the enhanced chemiluminescence method (Pierce). For transient transfection experiments SK-N-BE(2) cells were seeded 1 day prior to transfection with expression vectors coding for full length Notch-1 (f.l. Notch-1) or intracellular Notch-1 (i.c. Notch-1) (Aster *et al*, 2000), both kindly provided by Dr Jon Aster, USA. As a control for transfection the empty vector (pcDNA3) was used. In all experiments 4.8 *μ*g DNA and 9 *μ*l Lipofectamine 2000 (Life Technologies, Rockville, MD, USA) were used. The cells were then treated and harvested as described for Western blot analyses.

### RNA preparation and Northern blotting

The guanidine-isothiocyanate/phenol-chloroform extraction method was used to isolate total cellular RNA (Chomczynski and Sacchi, 1987). In total, 15 *μ*g RNA was separated on a 1% formaldehyde agarose gel and blotted onto Hybond-N nylon membrane (Amersham). A *neuropeptide tyrosine* (*NPY*) probe, generated as described previously (Bjelfman *et al*, 1990), was labelled with [^32^P]dCTP using an oligonucleotide labelling kit (Amersham).

### RNA purification and cDNA synthesis

Total RNA was purified with RNeasy kit (QIAgen, Hilden, Germany) as described by the manufacturer where after 2 *μ*g of RNA was incubated with 2 U RNase-free DNase (Promega, Madison, WI, USA). Samples were desalted and concentrated by centrifugation on Microcon-100 columns (Amicon, Millipore Corp., Cork, Ireland). For first strand synthesis 50 ng of pd (N) 6 random hexamers (AP Biotech, Freiburg, Germany), 5 × first strand buffer (Invitrogen), 2 *μ*l 0.1 M DTT (Invitrogen), 40 U Rnasin (Promega), 1 *μ*l 10 mM dNTP mix (AP Biotech) and 1 *μ*l superscript II were added to the purified RNA. The newly synthesised cDNA was diluted in a final volume of 500 *μ*l.

### Real-time quantitative RT–PCR

Real-time quantitative RT–PCR was performed by mixing 2 × master mix (qPCR Core kit for SYBR Green) (Eurogentec, Seraing, Belgium), 0.75 *μ*l C 1/2000 SYBR Green I, 250 nM of forward and reverse primer (Invitrogen) and RNase-free water to a total volume of 20 *μ*l. In total, 5 *μ*l of the cDNA template was added and the reaction mix heated to 95°C for 10 min. Amplification was carried out on an ABI5700 (Applied Biosystems) for 40 cycles with a denaturation temperature of 95°C for 15 s and annealing and extension temperature of 60°C for 1 min. DNase treated RNA was used as control of the purity of the samples. The genes analysed were: *NPY* (forward primer: 5′-TCCAGCCCAGAGACACTGATT-3′, reverse primer: 5′-AGGGTCTTCAAGCCGAGTTCT-3′); *GAP-43* (forward primer: 5′-ACGACCAAAAGATTGAACAAGATG-3′, reverse primer: 5′-TCCACGGAAGCTAGCCTGAA-3′); *GAPDH* (forward primer: 5′-TGCACCACCAACTGCTTAGC 3′, reverse primer: 5′-GGCATGGACTGTGGTCATGAG -3′)*; HPRT1* (forward primer: 5′-TGACACTGGCAAAACAATGCA-3′, reverse primer: 5′-GGTCCT TTTCACCAGCAAGCT-3′). The results were used to calculate relative quantity of expression of the different genes, using the reference genes *GAPDH* and *HPRT1* for normalisation. All experiments were performed in triplicates and the results were recorded as relative expression level.

### Luciferase reporter assay

SK-N-BE(2) cells were seeded in a 24-well plate 1–2 days prior to transfection. The cells were then transfected with 20 or 40 ng wild-type *Hes-1* promoter construct (kindly provided by Dr Kageyama, Kyoto University, Japan) (Nishimura *et al*, 1998) using Lipofectamine 2000 (Life Technologies). After transfection the cells were treated with either 2.5 *μ*M L-685,458, 1 or 2 mM VPA, or 50 or 100 nM TSA for 24 h where after cells were lysed and assayed for luciferase activity using the Dual-Luciferase Reporter Assay System according to the manufacturer's instructions (Promega). In control experiments equal volume of solvent was added, that is, methanol and/or DMSO respectively. Experiments were performed in replicates of three to six and repeated at least twice.

### MTT assay

The MTT assays were performed using the Promega CellTiter96® Non-Radioactive Cell Proliferation Assay kit according to the manufacturer's instructions. In short, drugs were added to a 96-well cell culture plate prior to seeding SK-N-BE(2) and SH-SY5Y cells. After 3 days, 10 *μ*l dye solution was added to each well and the plate was incubated for 4 h at 37°C, 5% CO_2_. 100 *μ*l Lys/Stop solution was added to each well and allowed to lyse the cells ON at RT. Next day, the absorbance was read using an ELISA microplate reader. Each concentration of the drugs was tested in triplicates. In control experiments equal volume of solvent was added, that is, methanol or DMSO, respectively.

### Flow cytometric analysis

For flow cytometric analysis of cell cycle distribution, both adherent cells and cells in the culture media were harvested by centrifugation, resuspended in 70% ethanol (−20°C) and stored at −20°C. Cells were then washed in cold PBS where after 800 *μ*l Vindelöv solution (3.5 *μ*M Tris (pH 7.6), 10 mM NaCl, 50 *μ*g ml^−1^ propidium iodide, 20 *μ*g ml^−1^ RNase, and 0.1% (volume for volume) NP-40) was added to the cells and left to incubate for at least 20 min on ice. DNA analyses were performed using a FACSCalibur flowcytometer (Becton Dickinson Immunocytometry System, San Jose, CA, USA), and the fraction of G1, S, G2/M and dead cells was determined using CellQuest 3.2 Software (Becton Dickinson Immunocytometry System). For assessment of apoptosis, an apoptosis detection kit labelling cells with FITC-conjugated annexin V was used as described by the manufacturer (R&D Systems). In short, cells were stimulated with VPA for 72 h where after cells were harvested, washed twice in cold PBS containing 2% BSA, and stained with FITC-conjugated annexin V for 15 min at RT. Cells were then washed and annexin V binding analysed by flow cytometry.

## RESULTS

### Expression of Notch-1, Hes-1 and Hash-1 in NB cells

**Figure 1:**
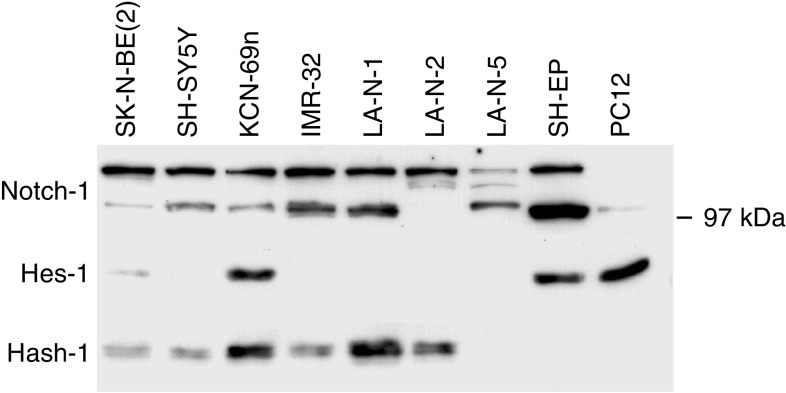
Protein expression analyses of Notch-1, Hes-1 and Hash-1 in NB cell lines. Cell lysates containing 50 *μ*g of protein from the human NB cell lines SK-N-BE(2), SH-SY5Y, KCN-69n, IMR-32, LA-N-1, LA-N-2, LA-N-5, SH-EP and the rat pheochromocytoma cell line PC12 were separated by SDS–PAGE. The proteins were blotted onto a PVDF membrane, which was probed with polyclonal anti-Notch-1 antiserum, polyclonal anti-Hes-1 antiserum or a monoclonal anti-Mash-1 antibody.

The expression patterns of Notch-1, Hes-1 and Hash-1 were analysed at protein level in NB cell lines and the rat pheochromocytoma cell line PC12 (Figure 1). All cell lines tested expressed varying levels of Notch-1 (Figure 1). The different bands represent the full-length protein (approximately 220 kDa) and the transmembrane protein (approximately 110 kDa). As has been shown previously (Jögi *et al*, 2002), all but two NB cell lines expressed Hash-1 (i.e. SK-N-BE(2), SH-SY5Y, KCN-69, IMR-32, LA-N-1 and LA-N-2). Hes-1 was detectable in SK-N-BE(2), KCN-69, SH-EP and PC12 cells. After longer exposure Hes-1 expression could be detected also in IMR-32 and SH-SY5Y cells (data not shown). During induced differentiation of NB cells we have previously reported a transient upregulation of Hes-1 accompanied by a decrease in the expression of Hash-1 (Grynfeld *et al*, 2000). Recent findings indicate that the transient activation of Hes-1 might be a direct consequence of an increase in Notch signalling activity (M Stockhausen, manuscript in prep.)

### VPA modulates the Notch signalling cascade in NB cells

**Figure 2:**
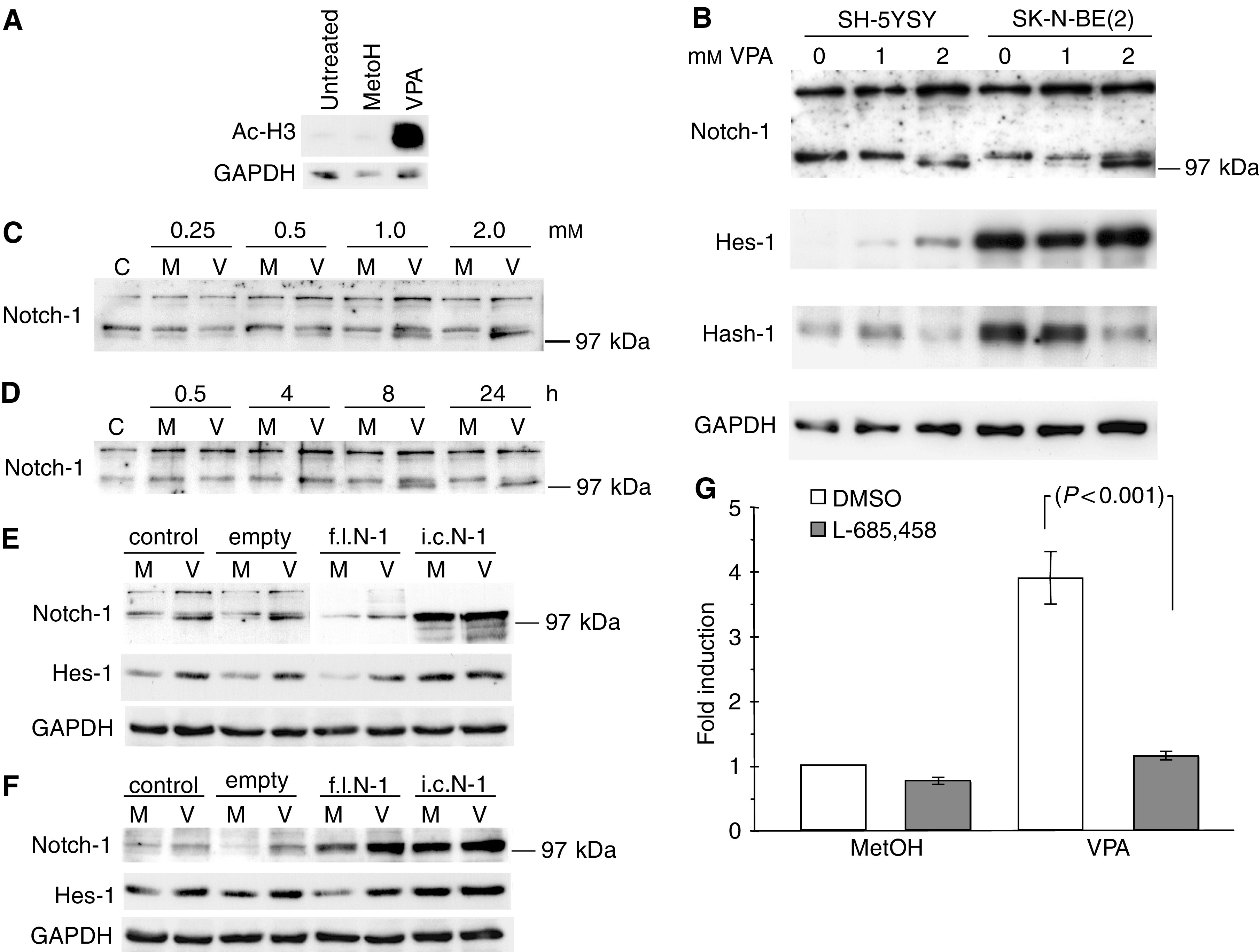
(**A**) Western blot analysis showing acetylation of histone H3 after treatment of SK-N-BE(2) cells with 1 mM VPA for 12 h. (**B**) Western blot analysis of Notch-1, Hes-1 and Hash-1 expression in SH-SY5Y and SK-N-BE(2) NB cell lines treated with 1 or 2 mM VPA for 72 h. (**C**, **D**) Western blot analyses of SK-N-BE(2) cells treated with increasing concentrations of VPA (V) for 24 h or with 1 mM VPA (V) for indicated times. Untreated cells (C) or cells treated with the solvent methanol (M) are included as controls. Notch protein expression was detected using an antiserum directed against the C-terminal domain of the receptor. (**E**, **F**) Western blot analyses of SK-N-BE(2) cells transfected with constructs expressing either full-length (f.l. N-1) or intracellular (i.c. N-1) Notch-1. In control lanes, extracts from untranfected (control) cells or extracts from cells transfected with the empty expression vector were used. The cells were treated with either 1 mM VPA (V) or the solvent methanol (M) for 24 h. In (**E**), antiserum against the C-terminal domain of the Notch-1 receptor was used. Since Notch-1 transfected cells express much higher levels of the Notch-1 protein compared to control cells, shorter exposure of these lanes is presented. In (**F**), an antiserum specifically reacting with the cleaved and hence activated form of the receptor was used. In addition, expression of Hes-1 was analysed. GAPDH was used as a control for equal loading of samples. (**G**) *Hes-1* promoter activity in SK-N-BE(2) cells after treatment with either VPA alone (1 mM) or in combination with the *γ*-secretase inhibitor L-685,458 (2.5 *μ*M). Cells were transfected with a vector containing the gene for luciferase under the control of the *Hes-1* promoter. The results are presented as fold induction over control cells and expressed as mean±s.d. of six replicates. Statistically significant changes were analysed by Student's *t*-test.

Next, we wanted to study whether it was possible to affect the Notch-associated differentiation of NB cells using HDAC inhibitors, since a functional Notch cascade relies on HDAC activity for a normal function of both RBP-J*κ* and Hes-1 (Grbavec and Stifani, 1996; Jimenez *et al*, 1997; Kao *et al*, 1998). We therefore treated NB cells with the HDAC inhibitor VPA. In a first experiment we confirmed the HDAC inhibitory effect of VPA by using an antisera directed against acetylated histone H3. As expected, treatment with 1 mM VPA for 12 h led to a dramatic increase in histone H3 acetylation (Figure 2A). Next, SH-SY5Y and SK-N-BE(2) NB cells were exposed to 0, 1 or 2 mM of VPA for 72 h and analysed for expression of proteins within the Notch signalling cascade. Hes-1 protein levels were increased, consistent with the view that *Hes-1* transcription normally is repressed by RBP-J*κ* in complex with co-repressors and HDAC, which in this setting was inhibited by VPA (Figure 2B). Even though the Notch-1 levels were unaffected by VPA, we noticed the appearance of a second faster migrating Notch-1 band with increasing concentrations of VPA, most likely representing the *γ*-secretase cleaved, and thereby activated form of Notch-1, i.c. Notch-1. Since the binding of i.c. Notch-1 to RBP-J*κ* has been proposed to be crucial for recruitment of the co-activating MAML complex (Kitagawa *et al*, 2001), the induction of i.c. Notch-1 would act in concert with reduced HDAC activity, further inducing the expression of Hes-1. Notably, Hash-1, which is repressed by Hes-1 in complex with Groucho and HDAC, was decreased upon treatment with VPA (Figure 2B). To further investigate the effect of VPA on Notch-1 activity we performed concentration and time course experiments. We could show that the appearance of the faster migrating Notch-1 band was dose dependent (Figure 2C). Already, at a relatively low concentration of VPA (0.5 mM), an accumulation of the faster migrating band was at hand and at 2 mM this band was the most prominent. Since it is known that HDAC inhibitors mediate their effects with rapid kinetics, we also performed a time course experiment showing that the faster migrating Notch-1 band started to appear already after 4 h and was clearly evident after 8 h of treatment with 1 mM VPA (Figure 2D).

In order to assess whether VPA treatment led to increased cleavage of the Notch-1 receptor, we transfected SK-N-BE(2) cells with a construct expressing full-length Notch-1 and treated the cells with 1 mM VPA. Using the C-terminal Notch-1 antisera we could detect an increase in the faster migrating band, indicative of increased cleavage of the overexpressed protein (Figure 2E). In control experiments using the *γ*-secretase independent i.c. Notch-1 construct no obvious changes in the level of the faster migrating band could be detected. To confirm the identity of the faster migrating band we also used an antiserum specifically recognising the S3 cleaved, and hence activated form of the Notch-1 receptor (Figure 2F). This experiment shows that the faster migrating band indeed is i.c. Notch-1, and that VPA enhances the processing of the full-length receptor, but has no clear effect on the expression of i.c. Notch-1. We could also detect an elevated expression level of Hes-1 in cells transfected with i.c. Notch-1, which was not further enhanced by the addition of VPA (Figure 2F).

**Figure 3:**
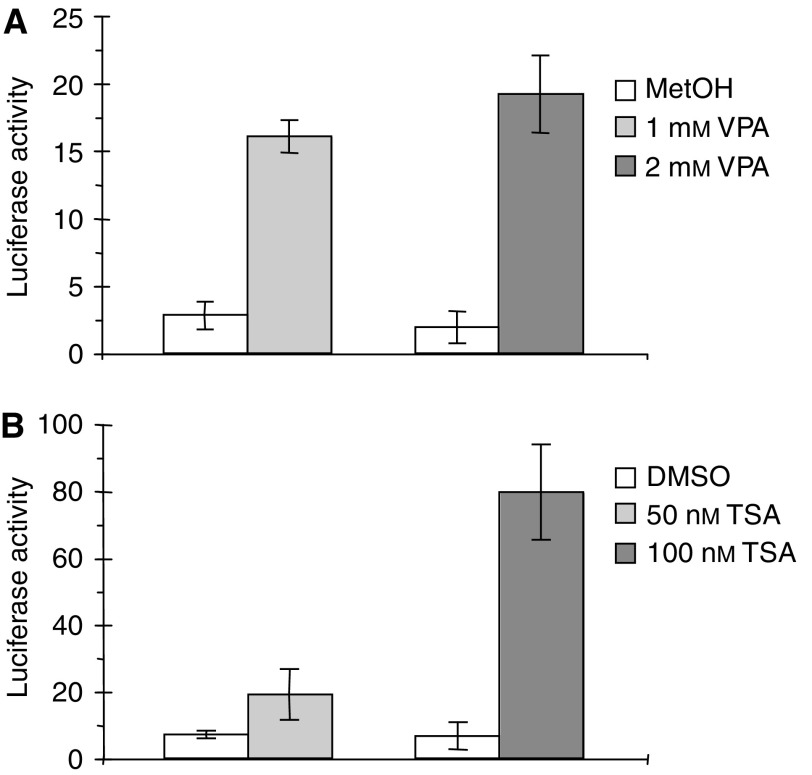
*Hes-1* promoter activity in SK-N-BE(2) NB cells treated with (**A**) VPA or (**B**) TSA. Cells were transfected with a luciferase reporter construct under the control of the *Hes-1* promoter. Each bar represents the mean of one triplicate. Error bars=s.d.

The *γ*-secretase inhibitor L-685,458 inhibits the activating cleavage (S3) of the Notch receptor and was therefore used to further dissect the effect of VPA on Notch-1 signalling activity. To obtain a quantifiable measurement of this effect we performed luciferase reporter assays using the *Hes-1* promoter. The cells were stimulated with VPA (1 mM) alone or in combination with L-685,458 (2.5 *μ*M) (Figure 2G). VPA treatment activated the *Hes-1* reporter whereas addition of L-685,458 significantly inhibited this induction, indicating that VPA induces a *γ*-secretase dependent activation of Notch signalling (Figure 2G). This notion was further strengthened in experiments where it was shown that VPA could enhance the reporter gene activation caused by co-transfection f.l. Notch-1 and that this effect could be partially repressed by addition of the *γ*-secretase inhibitor (data not shown). In addition, to investigate whether also other HDAC inhibitors affect the Notch signalling cascade, we performed *Hes-1* luciferase reporter experiments after treatment with either VPA or the well-established HDAC inhibitor TSA. Both treatments led to substantial activation of the *Hes-1* reporter construct in a dose-dependent manner (Figure 3). In summary, these results indicate that Notch signalling in NB cells is sensitive to HDAC inhibition and that VPA also seems to affect the processing of the Notch-1 receptor leading to increased levels of i.c. Notch-1.

### VPA decreases the proliferation of NB cells *in vitro*

**Figure 4:**
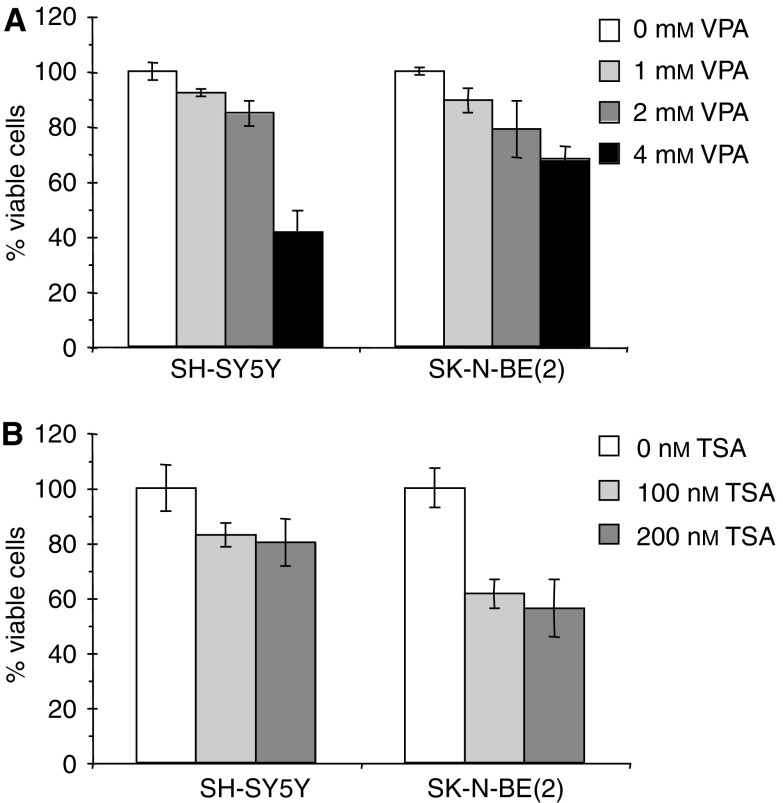
Cell viability analysis of NB cells treated with VPA or TSA. SH-SY5Y and SK-N-BE(2) cells were treated with the indicated concentrations of (**A**) VPA or (**B**) TSA for 3 days. The amount of viable cells was then assessed using MTT assay. The results are presented as a percentage of values after treatment compared to nontreated cells ±s.d.

**Figure 5:**
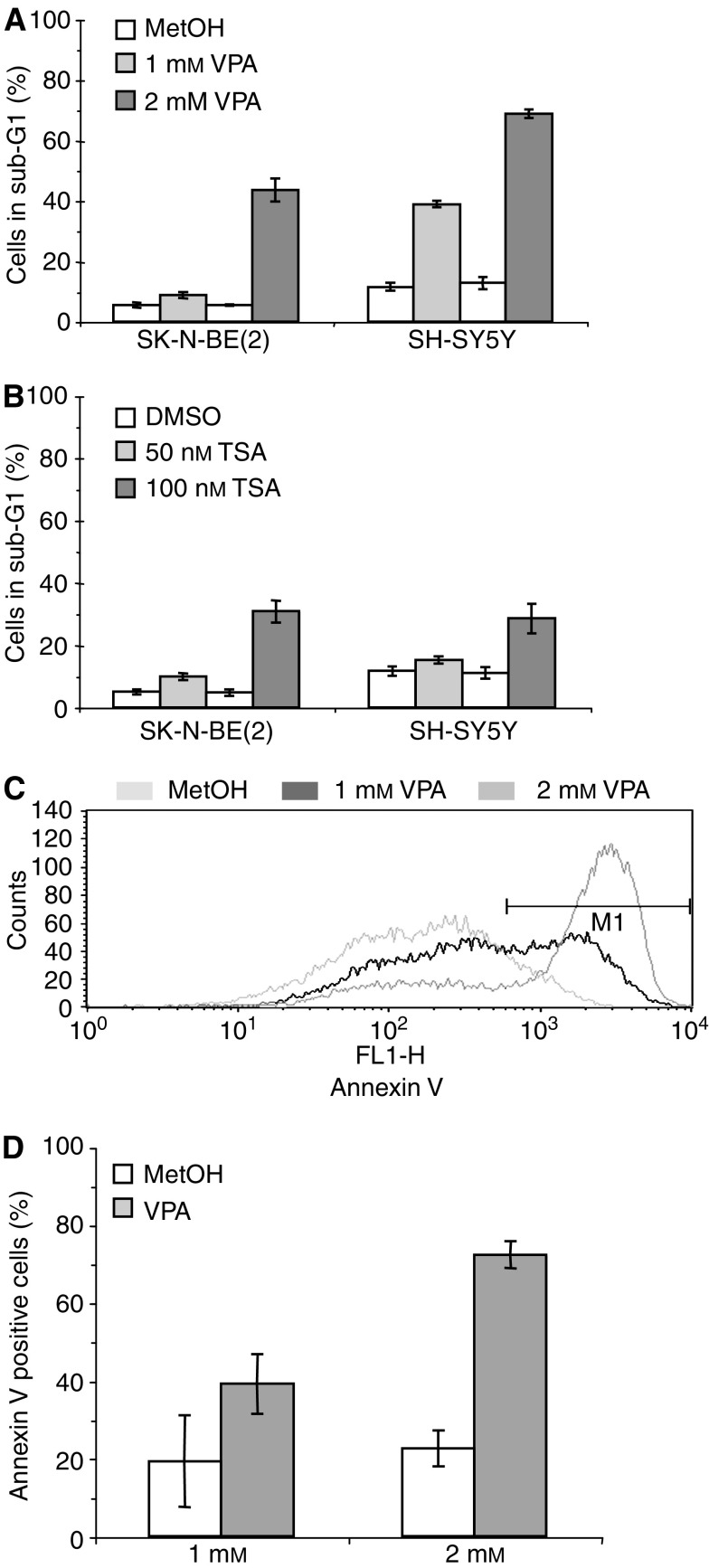
Flow cytometric analysis of dead cells after (**A**) VPA or (**B**) TSA treatment using propidium iodide staining. SK-N-BE(2) and SH-SY5Y NB cells were treated with VPA or TSA for 72 h where after both adherent and non-adherent cells were analysed. Control cells were treated with equal amounts of solvent, that is, methanol or DMSO, respectively. Experiments were performed in triplicates ±s.d. (**C**, **D**) Assessment of cell death using annexin V staining. Cells were cultured in the presence of VPA (1 or 2 mM) or solvent for 72 h and analysed by flow cytometry. Data are presented as a representative histogram (**C**) or as a quantification of the cells within the M1 gate (**D**).

Other studies have shown that VPA induces apoptosis and differentiation of NB cells both *in vitro* and *in vivo* (Cinatl *et al*, 1996, 1997). Based on these observations we performed MTT assays on SH-SY5Y and SK-N-BE(2) NB cells treated with VPA. With increasing amounts of VPA a decrease in the number of respiratory mitochondria, reflecting fewer viable cells, could be detected in the MTT assay (Figure 4A). Performing the same experiment with TSA, a similar decrease in the number of viable cells was observed (Figure 4B). This decrease could either be due to increased cell death or decreased proliferation, possibly as a result of induced differentiation. To discriminate between these two possibilities we performed propidium iodide stainings and FACS analyses of cell cycle distribution. No significant changes in cell cycle distribution could be detected (data not shown). When only attached cells were analysed no increased cell death could be detected (data not shown). However, when including the culture media in the analysis, treatment with VPA or TSA led to increased number of cells in the sub-G1 fraction, reflecting augmented cell death (Figure 5A and B). These results were further corroborated using FITC-conjugated annexin V in flow cytometric analyses. VPA treated cells showed a clear increase in annexin V staining, indicating increased apoptosis and/or cell death (Figure 5C and D). Thus, the decrease in the MTT assay was most probably primarily due to increased cell death. Further analyses of the attached VPA treated cells using TUNEL staining showed no increased apoptosis (data not shown). In summary, both VPA and TSA treatment led to increased cell death in a proportion of the cell population, in line with previous data (Cinatl *et al*, 1996, 2002). However, the cells that remained attached after treatment showed no increased apoptosis.

### VPA induces differentiation of NB cells *in vitro*

**Figure 6:**
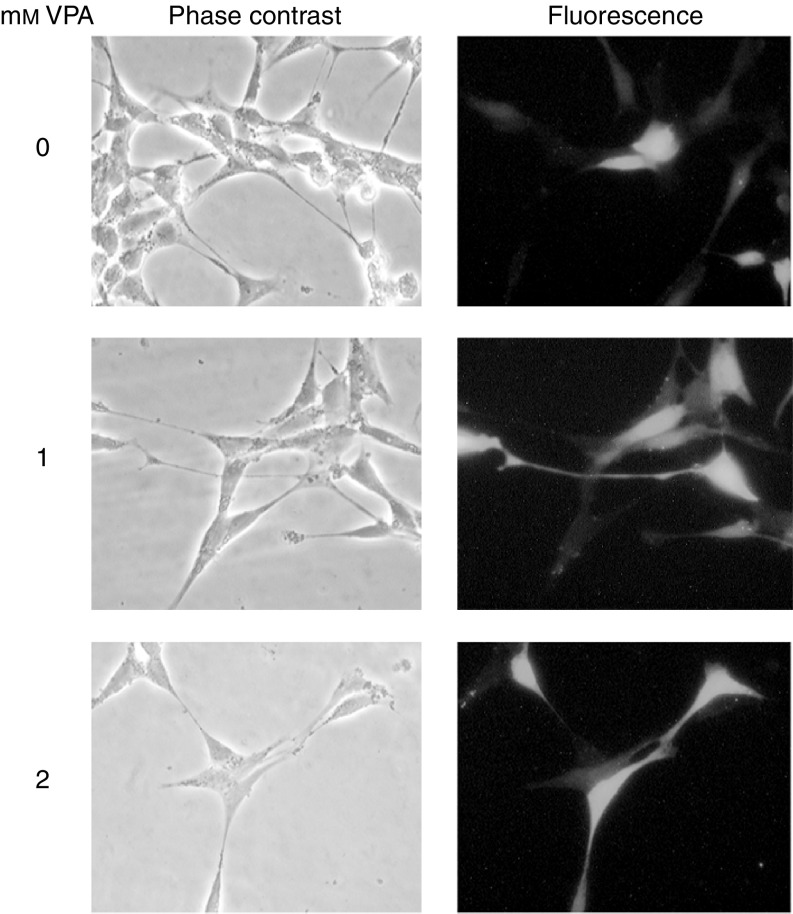
Morphology of VPA treated SK-N-BE(2) NB cells. SK-N-BE(2) NB cells were transfected with empty *pCMS-EGFP* vector and treated with 1 or 2 mM VPA for 72 h. Transiently transfected cells expressing EGFP were identified by fluorescence microscopy.

**Figure 7:**
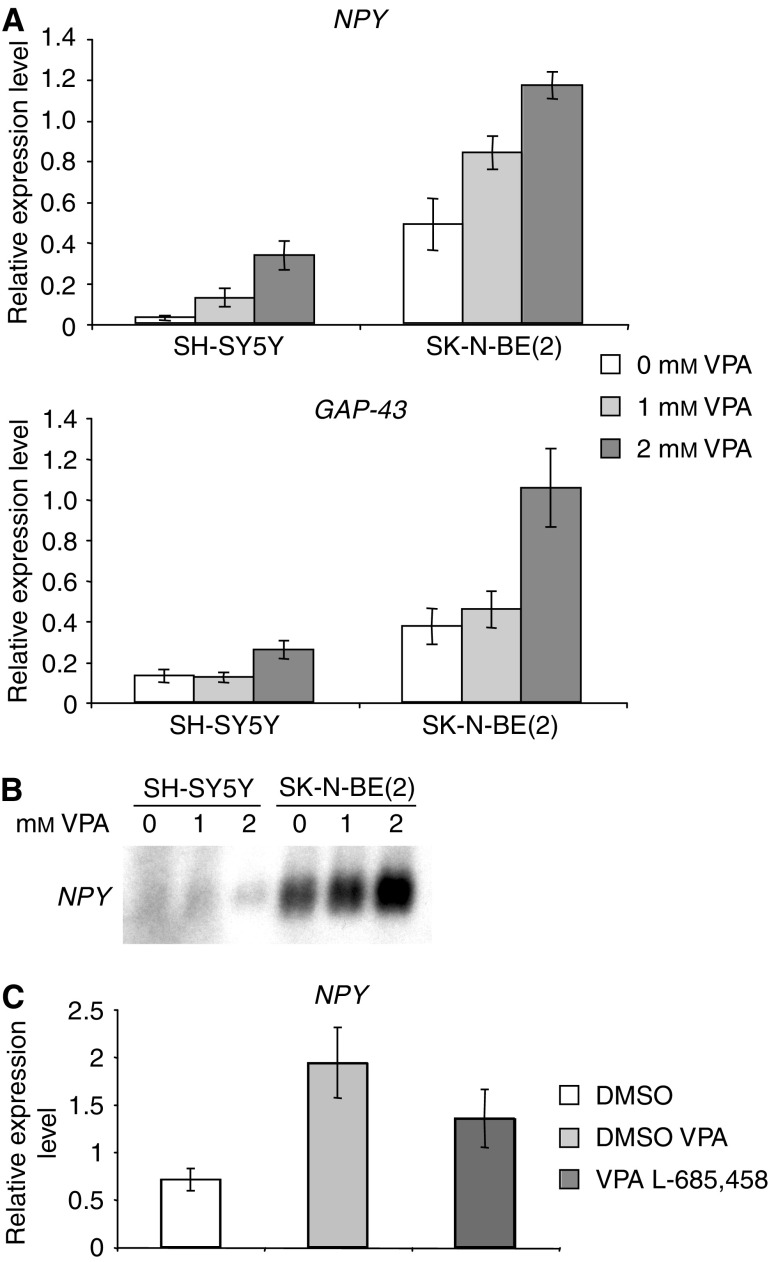
Differentiation status of VPA treated SH-SY5Y and SK-N-BE(2) NB cells. (**A**) Real-time quantitative RT–PCR analyses of NB cells treated with 1 or 2 mM VPA for 72 h showing *NPY* and *GAP-43* mRNA expression. Data are presented as relative expression levels using *GAPDH* and *HPRT1* as reference genes. Experiments were performed in triplicates ±s.d. (**B**) Northern blot analysis of total RNA (15 *μ*g) from SH-SY5Y and SK-N-BE(2) NB cells treated with 1 or 2 mM VPA for 72 h. Blotted filters were hybridised with a [^32^P]cDNA probe for *NPY.* (**C**) Real-time quantitative RT–PCR analyses of SK-N-BE(2) cells treated with 1 mM VPA in combination with 5 *μ*M L-685,458 for 72 h. Data are presented as relative expression level using *GAPDH* and *HPRT1* as reference genes. Experiments were performed in triplicates ±s.d.

With regard to the Notch signalling cascade, VPA treatment of NB cells led to changes similar to the ones observed when neuronal differentiation is induced by RA or TPA (Grynfeld *et al*, 2000; Jögi *et al*, 2002). In line with this notion, the morphology of VPA treated NB cells changed towards a more flattened phenotype with longer neurite extensions (Figure 6 and data not shown). To further characterise this phenotype we analysed the expression of some marker genes associated with neuronal/neuroendocrine differentiation using Northern blotting and real-time quantitative RT–PCR. A significant increase in *NPY* expression was detected using Northern blotting (Figure 7B). In the real time RT–PCR analyses this increase was shown to be several-fold (Figure 7A). Also, *GAP-43* expression was clearly induced when NB cells were treated with VPA (Figure 7A). These findings support previous reports by Cinatl *et al* (1997, 2002), suggesting that VPA induces both differentiation and cell death of human NB cells. Interestingly, the upregulation of *NPY* caused by VPA could be partially suppressed by the *γ*-secretase inhibitor L-685,458 (Figure 7C). This indicates that at least some of the differentiation-inducing effect of VPA might be a consequence of increased cleavage of the Notch receptor.

## DISCUSSION

Dysregulation of the Notch pathway has been implicated in several neoplasms (Allenspach *et al*, 2002). The consequences of such dysregulation does however seem to be highly cell type specific. For example, the constitutively activated form of Notch acts as a *bona fide* oncogene in leukaemia and in murine breast cancer, while recent data show that Notch-1 can function as a tumour suppressor gene in skin cancer (Ellisen *et al*, 1991; Uyttendaele *et al*, 1996; Nicolas *et al*, 2003). It has been suggested that these disparate effects might be dependent of the normal function of the cascade, that is, in tissues were Notch signalling is active in progenitor cells followed by a decreased signalling in differentiated cells, a constitutive activation of the cascade might lead to blocked differentiation or increased survival and proliferation (Allenspach *et al*, 2002). In other tissues, where Notch signalling is required for differentiation, exemplified by keratinocytes, loss of Notch activity might contribute to tumour development (Allenspach *et al*, 2002; Nicolas *et al*, 2003). Since Notch signalling is important during the embryonal development of the SNS, where it inhibits neuronal differentiation to take place prematurely, it seems likely that any dysregulated activity of this cascade would contribute to maintaining the cells in an undifferentiated, proliferating stage.

Neuroblastoma represents an almost unique tumour cell type in its capacity to differentiate upon treatment with different types of inducing agents, upregulating a well-established set of differentiation marker genes, making it possible to monitor the differentiation process (Påhlman *et al*, 1984, 1991; Bjelfman *et al*, 1990). We have previously shown that most NB cell lines express Hash-1 and that the expression of Hes-1 is transiently increased upon induced differentiation with a concomitant decrease in Hash-1 expression (Grynfeld *et al*, 2000). In this report we show that all NB cell lines express varying levels of Notch-1 (Figure 1). We, and others, have shown that Hes-1 can bind to the N-box in the *Hash-1* promoter and thereby repress Hash-1 transcription (Chen *et al*, 1997; Grynfeld *et al*, 2000). Since Hes-1 expression is under direct control of the Notch receptors, it is possible that these events are regulated by changes in Notch signalling activity during the differentiation process of NB cells. Our data suggest that during the initial phase of differentiation the Notch signalling cascade is activated. This leads to a rapid downregulation of Hash-1, accompanied by an upregulation of neuronal marker genes. These early events are followed by neurite outgrowth and cessation of proliferation. At this stage, Notch-1, Hes-1 and Hash-1 are all downregulated, possibly reflecting a terminally differentiated sympatho-adrenal phenotype.

We speculate that it can be possible to affect the proliferation capacity and differentiation status of NB cells by interfering with the Notch cascade. This could be achieved by specific interference with Notch activation. An alternative approach is to interfere with the intracellular components of the Notch cascade. Since transcriptional repression, in part mediated by HDAC activity, plays a pivotal role in the Notch cascade we have studied the effects of VPA on Notch signalling in NB cells. It has been known for two decades that VPA can inhibit tumour growth, and several preclinical studies on a wide variety of tumours have been performed (Blaheta and Cinatl, 2002). In a mouse NB cell line it was shown that VPA to a certain extent inhibits proliferation and VPA in combination with IFN-*α* led to neuronal differentiation of human NB cells (Cinatl *et al*, 2002). Furthermore, in xenograft experiments in mouse, VPA was shown to decrease tumour growth combined with a more differentiated phenotype of the NB cells (Cinatl *et al*, 1997, 2002). The mechanisms behind these effects remained elusive until recent findings showed that VPA is a potent HDAC inhibitor and that induced differentiation of carcinoma and leukaemic cells could be associated to this activity (Gottlicher *et al*, 2001).

It has been shown that VPA treatment of SH-SY5Y cells leads to activation of the MAP kinase signalling pathway, which in turn leads to induction of the AP-1 transcription factor (Yuan *et al*, 2001). Accordingly, several differentiation-related genes known to be activated by AP-1 was induced, such as *growth cone associated protein 43* (*GAP-43*) and *Bcl-2*. By using different inhibitors the authors could show that this effect of VPA was specifically mediated by the ERK pathway. This is of particular interest with respect to the effect of VPA on the Notch signalling cascade, since there seem to be a mutual influence between ERK signalling and the Notch pathway. For example, in T-cell leukaemia it was shown that Notch-induced transformation required the activation of MAP kinase and PI-3 kinase activity (Fitzgerald *et al*, 2000). Conversely, it was shown that activation of Notch signalling was an obligate step in Ras-induced transformation in an experimental system using human fibroblasts (Weijzen *et al*, 2002). However, we only detected very modest activation of the ERK pathway upon VPA treatment (data not shown). This discrepancy is probably due to the fact that we have performed our experiments during full serum stimulation. In our experimental set up it is therefore less likely that the dramatic effect on Notch signalling upon VPA treatment is a direct consequence of ERK activation. It is however known that Hes-1 is particularly sensitive to mitogenic stimulation and further studies are required to dissect whether some of the effects reported here in part might be a consequence of MAP kinase activation.

In this study we show that VPA causes changes of the Notch signalling cascade similar to changes detected during the early stages of TPA or RA induced differentiation of NB cells (Figure 2B) (Grynfeld *et al*, 2000). We noted increased levels of Hes-1 and decreased levels of Hash-1. The increased levels of Hes-1 could be due to the HDAC inhibiting effect of VPA, which would inhibit the repressive effect of RBP-J*κ* on *Hes-1* expression, or alternatively by affecting other associated factors such as MAML. The decreased level of Hash-1, on the other hand, might seem more paradoxical since Hes-1 is known to repress transcription from the *Hash-1* promoter by recruiting Groucho and HDAC, which is inhibited by VPA (Chen and Courey, 2000). It was however recently shown that Hes-1 could repress transcription of target genes by an additional, TSA insensitive, HDAC called SIRT. SIRT and Groucho/HDAC were proposed to operate synergistically since they bind different regions of Hes-1 (the bHLH domain and the C-terminal tetra peptide motif, respectively) (Takata and Ishikawa, 2003). Thus, the decrease in Hash-1 levels in the presence of the HDAC inhibitor VPA might be due to additional repression by SIRT. Hash-1 expression might however be regulated on several levels. For example, in small cell lung cancer it has been shown that Notch-1 induces degradation of the Hash-1 protein (Sriuranpong *et al*, 2002). Alternatively, the decreased Hash-1 expression after VPA treatment might solely reflect a more differentiated phenotype.

We also noted that VPA seems to affect the level of Notch-1 activation since we observed an accumulation of the activated form of the receptor, i.c. Notch-1 in Western blot experiments (Figure 2). We could show that this accumulation was rather rapid, with an increase in the level of cleaved Notch-1 starting already after 4 h and that it was concentration dependent. In addition, using cells transfected with *Notch-1* constructs we could show that VPA treatment led to elevated levels of the active form of the receptor. The mechanisms behind these findings are currently unclear. It might however be speculated that it involves increased activity of the *γ*-secretase complex. Using a *Hes-1* luciferase reporter assay we could show that VPA induced a substantial activation of the promoter and that this activation could be suppressed by the *γ*-secretase inhibitor L-685,458 (Figure 2G). Whether these results reflects an increased Notch signalling activity or alleviation of the HDAC-dependent repression of the *Hes-1* promoter remains to be determined. Thus, VPA might affect the Notch signalling cascade at different levels simultaneously.

The VPA induced changes in the Notch cascade described above, are similar to the changes in Notch signalling noticed during the initial phase of RA- or TPA-induced differentiation. This notion was substantiated by the findings that two well-established neuronal marker genes, *NPY* and *GAP-43* were upregulated and by changes in morphology (Figure 6 and 7). We suggest that these events reflect a partial induction towards a more differentiated phenotype. A clear increase in cell death was also noted upon treatment with VPA (Figures 4 and 5), in line with data presented in previous reports (Cinatl *et al*, 1997).

The Notch signalling cascade represents an appealing target for tumour cell differentiation therapy. We show here that the well-tolerated HDAC inhibitor VPA can be used to modulate the Notch signalling cascade in NB cells and that this can lead to induced differentiation together with increased cell death. These findings might explain some of the antitumorigenic effects of VPA.
